# The relationship between spiritual health and quality of life among COVID-19 patients with long-term complications in the post-coronavirus era

**DOI:** 10.3389/fpubh.2024.1371110

**Published:** 2024-05-30

**Authors:** Mehdi Safarabadi, Vahid Yousofvand, Ali Jadidi, Seyed Mohammad Taghi Dehghani, Kazem Ghaffari

**Affiliations:** ^1^Department of Nursing, Khomein University of Medical Sciences, Khomein, Iran; ^2^Student Research Committee, School of Nursing and Midwifery, Shahid Beheshti University of Medical Sciences, Tehran, Iran; ^3^Department of Nursing, Arak University of Medical Sciences, Arak, Iran; ^4^Department of Islamic Studies, Khomein University of Medical Sciences, Khomein, Iran; ^5^Department of Basic Medical Sciences, Khomein University of Medical Sciences, Khomein, Iran

**Keywords:** spirituality, spiritual health, quality of life, COVID-19, nursing, long term complications

## Abstract

**Objective:**

COVID-19 has varied manifestations and can cause complications that affect quality of life. Spiritual health may be a source of adaptation for these patients. This study investigated the relationship between spiritual health and quality of life among COVID-19 patients with long-term complications in the post-coronavirus era.

**Participants/methods:**

This study enrolled 475 COVID-19 patients through convenience sampling from medical facilities located in the Central Province of Iran. Data collection occurred between November 2022 and July 2023. A demographic checklist was utilized to ascertain the presence of potential COVID-19 complications. Patients exhibiting at least one long-term complication of COVID-19 were classified into the group with complications, while those without such complications were categorized into the group without complications. Subsequently, spiritual health and quality of life were assessed utilizing Paloutzian and Ellison’s Spiritual Well-Being Scale and the 36-item Short Form Health Survey (SF-36), respectively. Statistical analysis was conducted using SPSS-20.

**Results:**

The mean scores of spiritual well-being and quality of life for participants without COVID-19 complications were 70.87 ± 22.44 and 61.30 ± 18.33, respectively. In contrast, the mean spiritual health scores and quality of life for participants with COVID-19 complications were 41.20 ± 12.49 and 33.66 ± 1.46, respectively. Moreover, spiritual well-being was positively associated with quality of life among COVID-19 patients (*p* < 0.05).

**Conclusion:**

This study indicates that COVID-19 complications can impair patients’ spiritual health and quality of life, leaving them vulnerable and distressed. However, patients with higher spiritual health can cope better and enjoy a higher quality of life, despite challenges. Therefore, this study highlights the importance of addressing the spiritual needs of patients with COVID-19 complications and providing them with adequate support and care.

## Introduction

In December 2019, a novel and unique virus from the Coronavirus family, caused a viral disease outbreak in Wuhan, China. This disease, called COVID-19, has different symptoms but often affects the respiratory system (1). In Iran, as of March 2024, the cumulative number of COVID-19 cases has surpassed 7.6 million, with over 146,000 reported deaths attributable to the virus. A substantial portion of the population has undergone vaccination against the disease and adheres to preventive measures such as wearing masks and gloves, particularly during periods of heightened transmission (2).

Some patients may experience persistent or long-term complications arising from COVID-19. Among these, systemic, neuropsychiatric, cardiorespiratory, and gastrointestinal symptoms are frequently reported (3). Besides feeling tired for months after getting infected, long-term COVID-19 can cause various problems and consequences for patients (4).

There has been evidence that COVID-19 infection can have severe and lasting consequences for some patients. Fibrosis of the lungs, venous thromboembolism, arterial and cardiac thrombosis, inflammation of the cardiovascular system, stroke, dermatological complications, and depression are examples of these conditions (4). These long-term complications can vary depending on the patient’s characteristics and how long they have the symptoms (5). In some studies, the factors influencing long-term and multiorgan systemic COVID-19 have been examined. Many factors, including endothelial damage, disordered immune response, and hypercoagulability, often lead to thrombosis due to these factors (6). It is also possible that the immune system may attack its own cells in COVID-19 patients, similar to what happens in some autoimmune diseases (7).

The disease’s newness and the lasting symptoms cause more severe complications for patients who undergo this change (4, 8). The quality of life (QOL) of these individuals is negatively affected as a result. Even after they recover, clinically stable COVID-19 patients may still suffer from low QOL (9). To provide better support to those who have COVID-19, healthcare professionals, and government agencies need to know how it affects them (10, 11). QOL is a common measure for examining and judging one’s health and well-being.

It is important to understand that quality of life (QOL) is a multidimensional concept, reflecting aspects of physical, psychological, social, and spiritual well-being (12). Among these aspects, spiritual health is often overlooked, despite its importance for individual and social life. An individual’s spiritual health affects their health in a number of ways and is therefore crucial to their physical as well as social well-being. Furthermore, it also plays a major role in the enhancement and development of mental and emotional well-being. Most psychologists and specialists have stressed spiritual health in recent years (13, 14).

It is the belief of the World Health Organization that spiritual health is one of the most influential dimensions of human life. It is the fourth fundamental dimension of spirituality that should be promoted (15). Spiritual health is a new dimension of health in today’s world, besides physical and mental health. Increasing quality of life and giving meaning to all aspects of health are its benefits (16). In addition to helping people stay healthy, spiritual health prevents symptoms like depression, loneliness, and meaninglessness. Spiritual health is also related to reducing physical and mental diseases, especially in times of crisis, such as acute diseases (17).

The spiritual health of COVID-19 patients experiencing post-coronavirus complications may be associated with their overall quality of life. However, this aspect has received limited attention in existing literature, leaving many aspects of this relationship unexplored and warranting further investigation. This study sought to determine correlation between spiritual health and quality of life among COVID-19 patients experiencing long-term complications in the post-coronavirus period.

## Participants and methods

### Study design

A multicenter cross-sectional study was conducted.

### Participants

Based on the study by Kmita et al. (18) and considering *α*=/05 and the power of the study *β*=/1, also δ_1_ = 90, δ2 = 51, μ_1_ = 5.67, μ_2_ = 90, the sample size calculation yielded 443 individuals. Accounting for a 10% attrition rate, the final sample size was approximately 475, who were selected using convenience sampling. In this way, by referring to the files of patients infected with corona since the beginning of the outbreak of this disease, the people who had the conditions to participate in the study were selected as the sample size.


$$n=\frac{{\left(z_{1-\frac{\alpha }{2}}+z_{1-\beta }\right)}^{2}\left(\delta_{1}^{2}+\delta_{2}^{2}\right)}{{\left(\mu_{1}-\mu_{2}\right)}^{2}}$$

The Inclusion criteria for the study comprised individuals meeting the following conditions: a documented history of COVID-19 infection confirmed by positive PCR test results spanning at least the preceding 3 months, age above 18 years, and expressed willingness to participate in the study. Exclusion criteria encompassed individuals who declined to participate, lacked a verifiable medical history of COVID-19 infection, presented with one or more psychological disorders, or were below 18 years of age. Comorbidities that may impact spiritual health were not excluded in this study. Patients exhibiting at least one long-term complication associated with COVID-19 were classified into the group experiencing complications, while those without such complications constituted the comparison group. Long-term complications of COVID-19 may manifest across various physiological systems, including but not limited to the cardiovascular, respiratory, neurological and endocrine systems. This study was conducted from November 2022 to July 2023.

### Instruments

#### Demographic information questionnaire

In the demographic information questionnaire, participants were asked about their age, gender, field of study, marital status, education level, income, work experience, exposure to COVID-19 patients, underlying diseases (such as respiratory dysfunction, hematology disease, dermatology, hypertension, diabetes, heart disease, high cholesterol, hyperthyroidism, hypothyroidism, kidney failure, and others.), and mental health history.

#### Paloutzian and Ellison’s spiritual well-being scale (SWBS)

There is a questionnaire related to spiritual health that contains 20 items that assess two aspects of health (19). Existential health and religious health each consist of 10 questions. Existential health questions are even, while religious health questions are odd. It has a Likert scale with a score range of 20 to 120. There are three levels of scores in the questionnaire: low (20–40), average (41–99), and high (100–120). Farahani Nia in Iran validated this questionnaire (20). Among the findings of Seyedfatemi et al., it was determined that the validity of spiritual health was determined by the content validity, and that the reliability was determined by Cronbach’s alpha reliability coefficient, which was 0.82 (21). The questionnaire’s internal consistency for spiritual health, religious health, and existential health was obtained using Cronbach’s alpha method. The obtained values were 0.93, 0.91, and 0.92, respectively.

#### 36-item short-form health survey (SF-36)

SF-36 is a self-administered general instrument widely used to assess health-related quality of life. A total of 36 items in eight subscales comprise two generic components: physical component summary (PCS) and mental component summary (MCS). In the PCS, there are four subscales: physical functioning (10 items), role-physical (4 items), bodily pain (2 items), and general health perceptions (5 items). There are also four subscales in the MCS: social functioning (2 items), role-emotional (3 items), mental health (5 items), and vitality (4 items). A separate item examines individual health changes. The scores are then summed and converted to a scale of 0 to 100, with 0 representing the worst condition and 100 representing the best. Montazeri et al. established the validity of the SF-36 questionnaire in Iran over a span of 15 years. The questionnaire’s reliability was confirmed through Cronbach’s alpha coefficients ranging from 0.77 to 0.90 for its subscales and the overall scale (22). In this study The Cronbach’s alpha coefficient attained a score of 0.92, demonstrating favorable reliability.

### Ethical considerations

At the beginning of the study, researchers explained the study’s objectives and informed consent. Participants were assured that their information would remain confidential, and the results would be presented in a general manner. The Khomein University of Medical Sciences Ethics Committee approved the study protocol (IR.KHOMEIN.REC.1402.006).

### Statistical analysis

The collected data underwent analysis utilizing SPSS-20 software and descriptive statistical techniques, encompassing measures such as mean, standard deviation, frequency, and percentage distribution tables, to characterize the variables. Demographic variables were assessed using the Chi-square test. Analytical approaches, including Pearson’s correlation, were employed to evaluate the association between spiritual health and quality of life. Simple linear regression was utilized to investigate the relationship between spiritual health, quality of life, and unadjusted complications, while multiple linear regression was employed to control for potential confounding variables. The analyses were conducted at a significance level of 5%.

## Results

In this study, most participants were 41–60 years old. The majority of participants were male and had education levels below a diploma. Most of the participants were married and self-employed (Table 1).

**Table 1:** Participants demographic characteristics (*N* = 475).

Variable	type	*N*	Complication	No complication	*p*-value
Age, Mean ± SD	18–40	28	62.93 ± 1.50 *	58.24 ± 0.62*	< 0.001
	41–60	235			
	>60	212			
Gender, *n* (%)	Female	167	25	142	> 0.05
	Male	308	50	258	
Educational level, *n* (%)	Below a diploma	161	15	146	< 0.001
	Diploma	138	20	118	
	Bachelor	108	19	88	
	Master and above	68	21	48	
Marital status, *n* (%)	Married	356	48	306	> 0.05
	Single	119	27	94	
Jobs	Freelance	170	35	135	< 0.001
	Employee	109	22	89	
	Housewife	196	23	176	

* Mean and Std. Deviation.

The mean scores of spiritual health and quality of life for participants without COVID-19 complications were 70.87 ± 22.44 and 61.30 ± 18.33, respectively. The mean scores of spiritual health and quality of life for participants with COVID-19 complications were 41.20 ± 12.49 and 33.66 ± 1.46, respectively. The highest mean spiritual health scores and quality of life dimensions for participants without COVID-19 complications were existential health (35.79 ± 12.49) and general health (65.05 ± 21.44), respectively. The highest mean scores of spiritual health and quality of life dimensions for participants with COVID-19 complications were existential health (21.86 ± 6.38) and role-emotional (33.06 ± 12.03), respectively (Table 2). The study also showed that the person correlation coefficient between spiritual health and quality of life was 0.17, and it was statistically significant (*p* < 0.001) (Table 3).

**Table 2:** Mean scores of spiritual health and quality of life for participants with and without COVID-19 complications.

Variable	Total	Complication	No complication
	Mean ± SD	Mean ± SD	Mean ± SD
Spiritual health (Total score)	66.15 ± 23.78	41.20 ± 12.49	70.87 ± 22.44
Religious health	32.72 ± 11.74	19.33 ± 8.01	35.24 ± 10.58
Existential health	33.59 ± 12.79	21.86 ± 6.38	35.79 ± 12.49
SF-36 quality of life (Total score)	56.93 ± 20.24	33.66 ± 1.46	61.30 ± 18.33
Physical functioning	55.2 ± 22.88	30.33 ± 12.68	54.40 ± 19.18
Role-physical	59.210 ± 22.67	31.53 ± 11.11	64.45 ± 20.40
Bodily pain	54.90 ± 21.08	29.60 ± 13.21	59.65 ± 18.78
General health	59.92 ± 23.46	32.60 ± 12.13	65.05 ± 21.44
Vitality	58.49 ± 21.85	33.13 ± 12.04	63.25 ± 19.91
Social functioning	54.65 ± 23.26	32.80 ± 12.13	58.75 ± 22.56
Role-emotional	54.69 ± 22.48	33.06 ± 12.03	58.75 ± 21.66
Mental health	50.89 ± 24.58	31.60 ± 14.27	63.24 ± 21.56

**Table 3:** The correlation coefficient between spiritual health and quality of life of study participants.

Variable	SF-36 quality of life (N)	Pearson correlation	*p*-value
Spiritual health	475	0.17	0.00

Linear regression showed that the mean score of spiritual health for people who had COVID-19 complications was 29.63 points lower than for people who did not have COVID-19 complications (*p* < 0.001). Similarly, the mean score of quality of life for people who experienced COVID-19 complications was 27.63 points lower than for people who did not experience COVID-19 complications (*p* < 0.001) (Table 4).

**Table 4:** The linear regression of spiritual health and quality of life among participants with and without COVID-19 complications.

Variable	Unstandardized Coefficients		Standardized Coefficients		*p*-value*	Unstandardized Coefficients		Standardized Coefficients		*p*-value**
	*β*	Std. Error	Lower Bound	Upper Bound		*β*	Std. Error	Lower Bound	Upper Bound	
Spiritual health (total score)	29.63	2.66	11.10	34.88	<0.001	33.10	2.65	38.32	27.89	<0.001
Existential health	13.93	1.47	16.83	11.02	<0.001	14.55	1.51	17.52	11.58	<0.001
Religious health	15.90	1.28	18.43	13.37	<0.001	17.67	1.28	20.19	15.14	<0.001
SF-36 quality of life (total score)	27.63	2.21	31.97	23.28	<0.001	29.60	2.23	31.16	22.37	<0.001
Physical functioning	28.16	2.57	32.22	23.10	<0.001	29.07	2.59	32.16	21.98	<0.001
Role-physical	24.06	2.27	28.54	19.58	<0.001	25.12	2.29	27.62	18.62	<0.001
Bodily pain	30.05	2.26	34.50	25.59	<0.001	30.60	2.31	34.14	25.06	<0.001
General health	31.54	2.59	36.64	26.44	<0.001	34.45	2.55	37.46	27.43	<0.001
Social functioning	25.95	2.67	31.20	20.69	<0.001	26.70	2.73	30.06	19.33	<0.001
Role-emotional	32.91	2.42	37.67	28.15	<0.001	33.19	2.54	36.01	26.37	<0.001
Mental health	29.46	2.59	34.56	24.36	<0.001	29.80	2.62	32.96	22.64	<0.001
Vitality	30.11	2.37	31.24	25.63	<0.001	31.11	2.39	33.82	24.04	<0.001

*Unadjusted whit Age, Educational level and Jobs.

** Adjusted whit Age, Educational level and Jobs.

On the other hand, chi-square tests and independent t-tests showed statistical significance for age, education level, and occupation. After adjusting for these variables in the linear regression test, the study results showed that the mean spiritual health score for people with COVID-19 complications was 33.10 points lower than for people who did not have COVID-19 complications. Similarly, the mean score of quality of life for people who experienced COVID-19 complications was 29.60 points lower than for people who did not experience COVID-19 complications (Table 4).

## Discussion

This study found that participants without COVID-19 complications had higher spiritual health (moderate score) than those with COVID-19 complications (poor score), and this difference was statistically significant (*p* < 0.001). Nodoushan et al. (11) reported that spiritual health was directly related to stress reduction in pregnant women with COVID-19, which aligns with this study. Similarly (11), Papazoglou et al. (23) demonstrated that spiritual health and religious practices could help improve and reduce the complications of this disease (23), which also agrees with this study.

According to the current study, spiritual health was directly related to quality of life. As Margeti et al. found, COVID-19 patients with higher spirituality had a higher quality of life (24). Also, Cherblanc (9) found that spiritual health and quality of life are directly related to each other in a survey of 2,754 COVID-19 patients (9).

In addition, the study found that participants without COVID-19 complications had a higher quality of life (moderate score) than those with COVID-19 complications (poor score). According to Ferreira et al. COVID-19 complications, such as stress, anxiety, and physical discomfort, lower the quality of life of patients (25). Similarly, as a result of a review of 34 studies, Shanbehzadeh et al. (17) examined the common complications of COVID-19, such as fatigue, pain, reduced physical capacity, and mental health issues. It has also been shown that the quality of life in COVID-19 patients who experience complications decreases up to 3 months after the procedure (17), which is also in agreement with this study’s findings.

The present study indicated that participants’ age, education, and occupation influenced their spiritual health and quality of life. According to Jadidi et al. ‘s study on the spiritual health of older adults, their spiritual health is influenced by age and social class, including occupation and income (15). In addition, Cherblanc et al. found that income level, age, and social class were associated with spiritual quality of life in COVID-19 patients (9). Additionally, Varricchio et al. in “Evaluation of the Quality of Life at the Bedside,” claimed that quality of life was influenced by age, education, social status, diseases, and spirituality (26).

Physical and mental crises threaten or harm individuals or communities’ health, well-being, or safety. The COVID-19 pandemic is an example. This crisis can negatively impact the quality of life (QoL) of affected people, such as reducing their physical, psychological, social, and environmental well-being (17).

QoL is a subjective and multidimensional concept that reflects how people perceive and evaluate their lives in various domains. These domains include health, happiness, relationships, work, leisure, and spirituality. Objective and subjective factors, such as personal characteristics, life circumstances, expectations, values, and beliefs, influence QoL. QoL is important for human development, health promotion, and social policy (27).

Spirituality is a broad and complex concept that refers to the search for meaning, purpose, and transcendence in life. Spirituality can be expressed in various forms, such as beliefs, practices, experiences, values, and relationships. Multiple factors, such as culture, religion, personality, and life events, can influence spirituality (28). Spirituality is a significant aspect of QoL, as it can provide hope, strength, peace, and resilience in the face of physical and mental crises (14).

Recent studies have explored the relationship between spirituality and QoL among different populations, such as healthy adults, patients with chronic diseases, palliative care patients, and healthcare workers. These studies suggest spirituality is positively associated with QoL, especially in the psychological, social, and environmental domains. Spirituality can also enhance individuals’ and communities’ coping and adaptation skills during physical and mental crises by providing a sense of coherence, connectedness, and compassion (14, 15).

However, the association between spirituality and QoL in patients who have complications after COVID-19 infection is not straightforward, as it is influenced by various factors, such as the definition and measurement of spirituality and QoL, the type and severity of the crisis, the personal and contextual resources available, and the individual and cultural differences in spirituality and QoL (29). Therefore, more research is needed to understand the complex and dynamic interplay between spirituality and QoL in different settings and situations.

In conclusion, physical and mental crises in post-COVID-19 patients with complications are one of the factors affecting the QoL of individuals and communities, which spirituality may help to improve or maintain (9). Spirituality is a valuable resource for enhancing the well-being and resilience of people facing physical and mental challenges, as well as for promoting a holistic and humanistic approach to health care and social policy (30).

## Strengths and limitations

The study’s considerable participant pool represents a notable strength, enhancing its potential for generalizability. However, the reliance on self-report measures introduces the possibility of response bias, warranting cautious interpretation of the findings. Moreover, the distinctive cultural attributes of the participants necessitate careful consideration when extrapolating and applying the study’s results. In addition, the cultural characteristics of spiritual health measures may affect the generalizability of the findings.

## Future directions and conclusions

This study highlights the potential correlation between COVID-19 complications and the deterioration of patients’ spiritual health and quality of life, underscoring their vulnerability and distress. The significant association observed between spiritual health and quality of life underscores the importance of integrating spiritual dimensions into healthcare provision for COVID-19 patients with long-term complications. Therefore, authorities are recommended to enhance the comprehensiveness and appropriateness of care services while developing programs and strategies to bolster patients’ spiritual health. Given the pervasive influence of spiritual beliefs in Iranian society, further research is recommended to devise and implement spiritually-centered interventions aimed at enhancing the quality of life in COVID-19 patients with long-term complications. Additionally, future studies should explore the relationship between spiritual health and the extent of progress and improvements in patients’ quality of life. Furthermore, employing structural equation modeling and causal inference methodologies in future research endeavors could facilitate the development of theoretical models in this domain.

## Data availability statement

The raw data supporting the conclusions of this article will be made available by the authors, without undue reservation.

## Ethics statement

The studies involving humans were approved by Ethics Committee of Khomein University of Medical Sciences. The studies were conducted in accordance with the local legislation and institutional requirements. Written informed consent for participation in this study was provided by the participants.

## Author contributions

MS: Validation, Methodology, Writing – review & editing. VY: Writing – review & editing, Writing – original draft. AJ: Writing – review & editing, Writing – original draft, Methodology, Conceptualization. SMTD: Writing – original draft, Supervision, Methodology. KG: Writing – review & editing, Data curation, Conceptualization.
